# Colorectal Cancer and the Enigma Surrounding Non-Canonical Wnt Signaling

**DOI:** 10.3390/cancers18162618

**Published:** 2026-08-14

**Authors:** Katsuhiro Kita

**Affiliations:** Department of Biology, St. Francis College, Brooklyn, NY 11201, USA; kkita@sfc.edu

**Keywords:** non-canonical Wnt, colorectal cancer, Wnt-5a, ROR2, hypoxia, cancer-associated fibroblasts

## Abstract

Since the discovery of truncated mutations of adenomatous polyposis coli proteins in familial adenoma patients in 1991, the mechanism of cytosolic β-catenin regulation has been intensively studied, and now it is very well known that the central role of the canonical Wnt/β-catenin is in colorectal cancer. However, Wnt signaling is very complicated because of the presence of almost 20 Wnt ligand genes, six Frizzled seven-transmembrane receptors, and three LRP co-receptors. In addition, research in the past two decades illuminated the existence of the other Wnt signaling—non-canonical Wnt signaling (Wnt/PCP and Wnt/Ca^2+^ pathways), and an increasing number of studies have shown the potential role of non-canonical Wnt signaling in cancer recently. One of the well-studied Wnt ligands in non-canonical Wnt signaling is Wnt-5a. However, the role of Wnt-5a and non-canonical pathways in cancer is mosaic—i.e., it may involve tumor-promoting or suppressing pathways. In certain cancers, non-canonical Wnt signaling may mainly act as a tumor promoter, yet the results are very controversial in colorectal cancer. Elucidating the role of non-canonical Wnt signaling in colorectal cancer may be very important to further reduce the risk of colorectal cancer, especially in patients who do not carry truncated mutations of adenomatous polyposis coli. In this review, I would like to mainly discuss the apparent controversy surrounding non-canonical Wnt signaling in colorectal cancer, and I would like to point out a few potential reasons contributing to the mysterious roles of Wnt5a-initiated non-canonical signaling in colorectal cancer.

## Simple Summary

Colon cancer is still the second leading cause of death worldwide, as well as in the United States. Although the majority of colon cancer cases may have mutations in key molecules of one signaling pathway called the Wnt/β-catenin pathway, there is still a certain percentage of patients suffering from colon cancer through a different signaling pathway. Wnt family consists of many molecules and is also transmitted via the non-β-catenin pathway (non-canonical Wnt signaling). In this review, I would like to shed light on the unclear role of non-canonical Wnt signaling in colon cancer, with a particular focus on one of the Wnt, Wnt-5a.

## 1. Introduction

Despite a more than 50% decrease in the overall mortality of colorectal cancer (CRC) from 1970 to 2020, CRC still remains the second leading cause of cancer-associated death in the United States and the world [1]. In CRC, the role of the Wnt/β-catenin signaling pathway has been well-acknowledged as one of the critical pathways involved in tumorigenesis, and thus, has been well-investigated. In canonical Wnt/β-catenin signaling, β-catenin is destroyed by ubiquitin-mediated proteolysis [2]. Regarding CRC, sporadic adenomatous polyposis coli (APC) truncated mutations occur in more than 80% of patients [3] and thus prevent the degradation of β-catenin, leading to the accumulation of cytosolic β-catenin.

**Figure 1:**
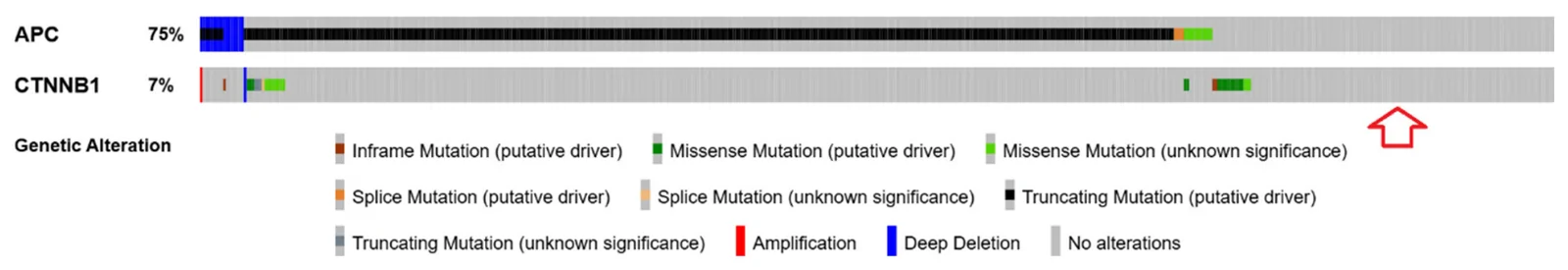
The alignment of APC and β-catenin alternations in the Cancer Gene Atlas’s colorectal adenocarcinoma data (PanCancer Atlas). The red arrow indicates the patient population that does not carry either APC or β-catenin alternations.

The first series of studies connecting Wnt signaling to cancer dates back to the early 1990s, performed by Dr. Harold Varmus’ lab and colleagues [4]. Those studies demonstrated that Wnt1 can promote tumorigenesis in some way. Around the same time, mutations of one of the key regulators in Wnt/β-catenin signaling, APC, were discovered for the first time [5,6]. Since then, the significance of APC mutations has compelled scientists to investigate their key role in tumorigenesis through β-catenin regulation [7,8,9]. Even without truncated APC mutations, β-catenin accumulations can occur; such cases involve β-catenin mutations [10]. However, there are still patients who do not carry either APC or β-catenin mutations. Figure 1 shows that a small fraction of colorectal adenocarcinoma patients (TCGA, PanCancer Atlas) do not carry APC mutations but β-catenin mutations (mutual exclusivity: *p* = 0.025). More importantly, although APC mutations are found in the majority of CRC patients, there are still over 20% of patients without APC and β-catenin mutations (Figure 1). The same trend is seen in metastatic CRC data by Memorial Sloan-Kettering Cancer Center (1134 samples [11]). This example implies that there are other signaling pathways independent of the Wnt-β-catenin signaling pathway. One such potential candidate would be the non-canonical Wnt signaling pathway [12].

Recently, non-canonical Wnt signaling has also been getting a spotlight in other areas, such as immunology [13] and cardiovascular diseases [14]. There are complex interplays between the canonical and non-canonical Wnt signaling pathways, as depicted well in more recent reviews [15]. In the area of cancer, we started seeing research reporting on non-canonical Wnt signaling and cancer since the early 2000s, yet approximately only 1/6 of studies compared the canonical Wnt signaling (search “canonical Wnt” and “cancer” as keywords to narrow down papers in PubMed, then compare it with “non-canonical Wnt” and “cancer” search) described non-canonical Wnt signaling in cancer. This may be partly because of the complexity of non-canonical Wnt signaling pathways compared to the canonical Wnt signaling pathway. In addition, many Wnt ligands (19~20 Wnt genes when you count A/B variants), 10 Frizzled receptors, and LDL receptor-related proteins (LRP) 1/5/6 are found in humans [14], making the focused analyses even more challenging. Besides Wnt receptors Frizzles, and co-receptor LRPs, other Wnt receptors in non-canonical pathways exist (receptor kinases, ROR1/2, and RYK). About receptors, I will focus on ROR1 and ROR2, because these two of the receptor kinases appeared to be the best-established cell surface receptors in the non-canonical Wnt pathway.

Regardless of the above complexity, the role of non-canonical Wnt signaling has been highlighted more during the past decade. Although few studies have clearly pinned down the crucial role of non-canonical Wnt signaling in CRC, the number of studies has been slowly increasing. Comparison of canonical and non-canonical Wnt pathways was recently reviewed well in a previous volume of *Cancers* [16]; therefore, I will not spare pages for redundant topics. Instead, this review aims to spotlight controversies surrounding non-canonical Wnt ligands, particularly Wnt-5a, in their role in tumorigenesis, which is probably independent of APC mutations or β-catenin accumulation. Because there are numerous review articles introducing non-canonical Wnt pathways (Wnt/PCP (planar cell polarity) and Wnt/Ca^2+^ pathways, such as [17]), I would like to focus on the major Wnt ligand that plays an important role in non-canonical pathways (focusing on Wnt-5a, Wnt-9a, and Wnt-11, with specific examples in CRC and other cancers). Then, I would also like to highlight non-Frizzled type receptors, ROR1/2. In the text, I will compare the current understanding of non-canonical Wnt pathways in CRC with those in other cancers. There are a few review articles focusing on Wnt-5a in CRC [18,19]. This review would highlight the tumor-suppressive role of Wnt-5a as well as little-known Wnt-5a isoforms. Additional insight, including the effect of different tumor stages, will be discussed; these will include the interplay between CRC and cancer-associated fibroblasts (CAFs) and hypoxia induction of non-canonical Wnt ligands. About the major kinase receptors, ROR1 and ROR2, I will also discuss the structural aspect of the ligand-binding domain, citing recent studies. I will also introduce the most recent studies that addressed hypoxia, which may solve the controversies about the role of Wnt-5a in CRC. Lastly, Section 4 lists and summarizes current challenges to point out the areas that would require further studies.

## 2. Non-Canonical Wnt Pathway

The first non-canonical Wnt pathway is the Wnt/PCP pathway. The Wnt/PCP pathway is involved in many physiological processes, such as zebrafish development [20], the establishment of cell orientation [21], lung alveolar repair [22], hair cell development [23], and even the spreading of Wnt in vertebrates [24]. One key molecule in the Wnt/PCP signaling pathway is the Dvl-associated activator of morphogenesis 1 (DAAM1). DAAM1 plays a critical role in Wnt-induced cytoskeletal rearrangement, as its activation is linked to Rho family small GTPases [25,26]. DAAM1 was also discovered as an effector for profilin [27]. DAAM1 is one of the formin family proteins [28]. Thus, it is not surprising to see the critical role of DAAM1 in developmental stages. Recently, DAAM1 knockout mice were embryonically lethal. In contrast, DAAM2 knockout was not sufficient for fetal survival, strongly suggesting a more crucial role of DAAM1 during development [29].

Recently, an increasing number of studies have shown the correlation between DAAM1 and cancer. For example, DAAM1 overexpression was reported to promote gastric cancer [30]; on the other hand, downregulation of DAAM1 expression by a microRNA, miRNA-137, suppressed esophageal cell carcinoma [31]. Studies focusing on the role of DAAM1 in other cancers are slowly expanding. For example, there are recent studies showing DAAM1 expression in breast cancer [32,33], and YWHAZ, a new downstream effector interacting with DAAM1, was reported [34]. DAAM1 is also involved in pseudopodia formation in breast cancer [35]. DAAM1 inhibition reduced cancer cell migration and proliferation in both breast and lung cancer models [36]. Note that the elevated level of DAAM1 is correlated with a poor clinical outcome [32], and DAAM1 inhibition was achieved by a small chemical molecule, etoposide [36]. Thus, taken together with a recent paper [30], increased expression of DAAM1—and possibly activation by Wnt/PCP pathway ligands—appears to be a promoting factor in tumor progression in CRC, particularly metastasis, which requires active cytoskeletal rearrangement of tumor cells as well as invasion into the extracellular matrices.

In regard to CRC, there is so far only one recent report showing the involvement of DAAM2, which is the paralog of DAAM1, in metastasis by activating matrix metalloproteases and PAK1, an effector of Rac [37]. However, overall, there are not many studies reporting the impact of DAAM1 on gastric cancer and CRC.

Besides the DAAM/Rho pathway, the Wnt/PCP pathway can also transmit the signal via the other Rho family GTPase, Rac1, as initially suggested more than two decades ago [38]. In mammalian cells (HEK293 = human), it was shown that the Rac pathway activates Jun kinase (JNK) independent of Rho [39]. Contrary to the above study, an earlier study using different mammalian cells (COS-7 = African green monkey), none of the Rho family GTPases were necessary to activate JNK in the Wnt/PCP pathway. To date, there are only sporadic studies dissecting the involvement of Rac1 in the Wnt/PCP activation of JNK. Thus, the Wnt-PCP pathway via Rac1 to the downstream JNK has not been clearly understood yet. Originally discovered as a UV-induced stress-responsive kinase related to apoptosis [40], JNK’s role in tumor necrosis factor-induced apoptotic signaling was well-established in the early 2000s [41,42,43]. However, as recent studies have shown, JNK is associated with CRC growth through a histone deacetylase Sirtuin 1 [44] and invasion through other signaling proteins such as calmodulin-regulated spectrin-associated protein 2 [45] and brain and muscle ARNT-like protein 1 [46]. In addition, the other study combined conditional knockout mouse strains emphasized a critical role of JNK activity in CRC stem cells, although I would be cautious about discussing this result in relation to the non-canonical Wnt signaling because the study used *Apc^Min^* mice, meaning that one of the APC alleles is truncated and thus may alter the canonical Wnt/β-catenin signaling. As you can see here, what has not been clarified is (i) the molecule connecting the non-canonical Wnt receptor to Rac (if not DAAM1 or DAAM2), and this should be a guanine exchange factor if Rac activation (=GTP form) is happening, and (ii) the effector molecule linking activated Rac to JNK in Wnt/PCP.

Compared with the Wnt/PCP pathway, there are even fewer studies that elucidate the role of the Wnt/Ca^2+^ pathway in CRC [47], and further studies are needed to determine the significance of Wnt/Ca^2+^, especially Wnt-5a/Ca^2+^, in CRC.

### 2.1. Wnt-5a in Cancer

As mentioned before, there are almost 20 Wnt ligands, making Wnt signaling very challenging to study [14]. One dilemma for us is that the specificity of each Wnt ligand does not bind like 0 or 100 to canonical and non-canonical receptors (In addition, there are 10 Frizzled receptors and three LRP co-receptors). Despite these extreme complexities, a few of the Wnt ligands showed more involvement than others in non-canonical Wnt signaling in cancer [48]. Among Wnt-5a, Wnt-7a, and Wnt-11, it became obvious that Wnt-5a has been best studied for its relevance to cancer [48].

To my knowledge, the first report showing the correlation between elevated Wnt-5a and gastric cancer was published in 2002 [49]. The same authors also reported higher Wnt-5a expression levels in breast cancer cells [50]. Soon, the Trent lab of the NIH reported the possible connection between Wnt-5a and metastatic melanoma [51]. The positive correlation between Wnt-5a and solid tumors was reported in breast cancer [52], osteosarcoma [53,54], neuroblastoma [55], lung [56], pancreatic cancer [57], prostate cancer [58], ovarian cancer [59], and hepatocellular carcinoma [60]. The initial finding by Saitoh was confirmed with a clinical study analyzing 237 cases of gastric cancer, and it was shown that approximately 30% of cases were Wnt-5a-positive [61]. As we can imagine from the potential link between the Wnt/PCP signaling and cancer, Wnt-5a may activate the Wnt/PCP pathway to promote tumorigenesis. It is very important to think about the connection between Rho family GTPase activation and cell motility, which was very well-proposed in a classic review [62]. Thus, it makes sense that the Wnt/PCP signaling activation can also promote metastasis of tumor cells. Besides the positive correlation of Wnt-5a upregulation in a variety of solid tumors, Wnt-5a was also reported to promote epithelial–mesenchymal transition in gastric [63] and pancreatic cancer [64].

However, in CRC, the situation may be puzzling. As earlier studies already indicated, the downregulation, but not the upregulation, of Wnt5a might be correlated with the progression of CRC [65]. Thus, the effect of Wnt-5a on tumor progression may be apparently the opposite between gastric cancer and CRC. Please note that there is a clinical study reporting a correlation between Wnt-5a promoter methylation and improved clinical responses/progression-free survival with 5-fluorouracil-based chemotherapy [66]. The review by Tufail and Wu nicely highlighted this “double-edged sword” role of Wnt-5a in CRC well [67].

Importantly, Wnt-5a expression apparently promoted tumor growth in non-small cell lung cancer [56]. Wnt-5a expression showed a correlation with poor clinical outcomes, possibly by promoting metastasis of melanoma [68]. However, this may not be the case in CRC. As briefly mentioned before, so far, the downregulation of Wnt-5a may lead to worse consequences in CRC [65,69]. Nevertheless, we have to very carefully dissect the role of Wnt-5a in CRC, because a recent report demonstrated CAF activation that potentially promotes CRC [70]. Thus, the role of Wnt-5a may be a double-edged sword in CRC progression [67], yet, the direct action of Wnt-5a through receptors may be more likely a tumor suppressor in CRC. If Wnt-5a activation of CAFs [70] becomes a solid story, then Wnt-5a’s action on CRC would largely depend on the stages of CRC as well because colorectal epithelial cells would interact with CAFs after adenoma formation or invading the basal membrane (see Discussion). As described in a review [71], the accumulation of CAFs is associated with higher grades of malignancy.

### 2.2. Wnt-5a as a Tumor Suppressor in CRC

As demonstrated in the past, Wnt-5a is indispensable for normal developmental processes, including body patterning, such as limbs and body formations [72,73,74]. One big question was whether or not Wnt-5a functions as a tumor suppressor [75,76,77,78,79] or a promoting factor; loss of Wnt-5a is reported to correlate with 44% of invasive ductal carcinomas [80], for example. In CRC, basically, Wnt-5a has been reported to function as a tumor suppressor. Wnt-5a promoter methylation or downregulation has been reported in CRC [65,81,82]. In addition to the downregulation of Wnt-5a by promoter methylation, upregulation of Wnt-5a was also reported to increase the expression of a tumor suppressor, 15-hydroxyprostaglandin dehydrogenase, in CRC [83]. A large-scale clinical study also supports the idea of Wnt-5a as a tumor suppressor in CRC; Wnt-5a promoter methylation was reported to be associated with microsatellite instability and B-Raf V600E mutation in a large cohort (over 1200) of CRC patients in Canada [84].

One study published last year, utilizing patient-derived organoids, described a possibly new molecular pathway underlying the tumor-suppressive effect of Wnt-5a via the transforming growth factor-β/NOTUM/OLFM4 pathway [85]. Thus, the role of Wnt-5a as a tumor suppressor may also be true in the 3D environment. In Section 2.1, I introduced the tumor-promoting effect of Wnt-5a via CAF [70]. Note that the patient-derived organoids may be devoid of CAF in culture. These controversies will be revisited in Section 2.3 and Section 4 to further discuss the details.

### 2.3. Wnt-5a Expression During CRC Progression

Whether or not Wnt-5a can be further complicated by the stage of CRC will be discussed. There was a new study utilizing a sophisticated treatment control model combined with Wnt-5a knockout mice [86]. This study also utilized single-cell RNA-seq (scRNA-seq) to get a clearer picture of CRC in this model system, because CRC is known to have four subtypes, as accepted in the community [87,88]. The detail and its significance will be revisited later (see Section 4).

### 2.4. Key Finding: Wnt-5a Isoforms

**Figure 2:**
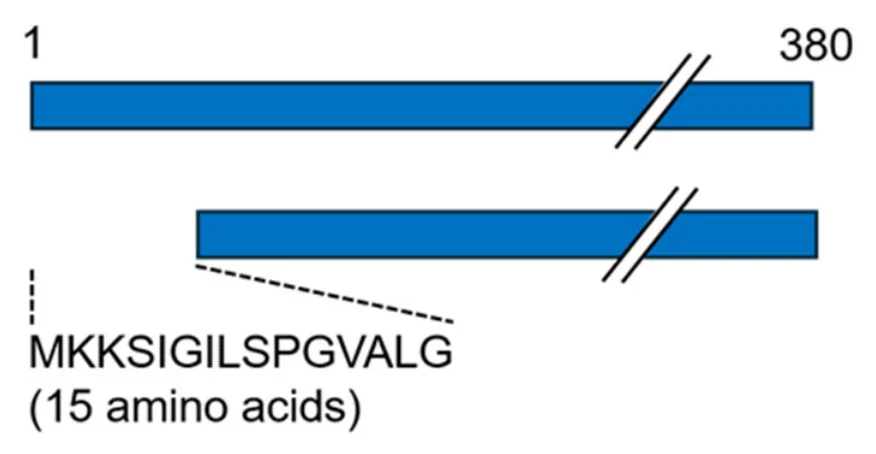
The schematic illustration of human Wnt-5a isoform 1 (longer form; (**top**), NM_003392.7) and Wnt-5a isoform 2 (shorter form; (**bottom**), NM_001256105.1) alignment. Both isoforms are completely identical except for the additional 15 amino acids on the N-terminus of isoform 1.

Although this topic may not have been discussed frequently, it might be potentially very important to understand the apparent controversy in the effect of Wnt-5a across different types of cancer. There are excellent review articles describing the role of Wnt-5a in cancer signaling [67,89]. I would like to point out one potentially interesting fact that may not have been discussed in the past review articles. Two Wnt-5a isoforms were reported a decade ago [90]. A year before this study, the structure of both mouse and human alternative promoters was clarified [91]. In the study by Bauer and colleagues [90], the authors discovered that there were three potential Wnt-5a variants. The longer version encodes the 380 amino acids of the Wnt-5a precursor, which is the Wnt-5a that most Wnt-5a studies focus on. On the other hand, three other variants, which utilize the alternative exon 1, produce slightly shorter (365 amino acids) variants. These differences, 15 amino acids in length at the N-terminal of the Wnt-5a protein, may not induce drastic structural changes. Figure 2 shows the schematic diagram of both isoforms.

It is very interesting to note that the longer Wnt-5a (Wnt-5a-L) inhibited the proliferation of tumor cells [90]. In this study, a triple-negative breast cancer cell line, MDA-MB-231, was used—a comprehensive investigation of the tumor-suppressive role of the longer form in other tumor cell lines would be beneficial to solidify this finding.

Supporting my idea, the distinct role of Wnt-5a isoforms in CRC was reported by Huang and colleagues [92]. According to this study, higher expression of the shorter Wnt-5a isoform and lower expression of the longer isoform are associated with clinical CRC progression, yet the clinical data presented in this study [92] may require a more careful look. Further investigations by a larger cohort of different ethnic groups in the future would be very informative.

### 2.5. Wnt-7a

A few reports have shown the involvement of Wnt-7a in CRC. Both reports essentially reach the same scientific point―a high level of Wnt-7a promotes CRC. The first report, back in 2002, used cell lines [93], and a recent study included patient samples analyzed with immunohistochemistry [94]. We should be very careful about the interpretation of these results, because cell lines used in these studies carry either APC mutations (HT-29 [95] and SW480 [96]). HCT116 expresses full-length APC; however, its β-catenin mutation makes it resistant to degradation [96]. At the same time, there was a report demonstrating the opposite trend [97]. Remarkably, the study examined 393 patients (all of whom suffered from colorectal adenocarcinoma). Disease-free survival and overall survival of Wnt-7a-negative patients in this study were approximately 70 months and 100 months, respectively (Wnt-7a-positive patients’ disease-free and overall survival were approximately 65 and 75% after the 120-month follow-up period (end point of the study). Thus, this clinical study with a relatively large cohort shared a clear benefit of Wnt-7a expression as a tumor-suppressive molecule in CRC. However, compared to the other Wnt ligands, there is clearly little research showing the role of Wnt-7a in CRC progression. Thus, further studies may unveil an unknown role of Wnt-7a in CRC.

Historically, Wnt-7a was shown to be a dorsal ectoderm-specific gene found during limb development in chicken [98] and mouse [99]. Therefore, the role of Wnt-7a in body patterning may be the main topic studied in the 1990s. The very first report of human Wnt-7a cDNA isolation was published in 1997 [100]. The Drabkin lab conducted very focused research showing that Wnt-7a expression disappeared in lung cancer but not in the normal lung epithelial cells [101]. This was soon supported by the fact that Wnt-7a induced E-cadherin expression via a β-catenin-specific molecular mechanism [102]. Primary tumor samples showed Wnt-7a downregulation, and Wnt-7a induction was demonstrated to mitigate the transformation potential of a panel of non-small cell lung cancer cell lines [103]. Its downstream mediator is extracellular regulated kinase-5 signaling [104]. The same trend was also observed in clear renal cell carcinomas [105] and estrogen receptor-positive breast cancer [106].

In ovarian cancer, the initial report described an opposite effect of Wnt-7a [107,108,109]. This line of evidence seems to be well-supported because less tumor development was observed when a Wnt-7a knockdown ovarian cancer xenograft model experiment was performed [110]. Furthermore, Wnt-7a signaling was shown to induce fibroblast growth factor-1 (FGF-1), a xenograft experiment using FGF-1 knockdown cells demonstrated reduced tumor size, and the study clearly showed that FGF-1 transcription is the direct target of Wnt-7a via the β-catenin-TCF pathway [111]. In bladder cancer, Wnt-7a was shown to promote invasion, and this is suppressed by miR-370-3p, a microRNA that represses Wnt-7a expression [112].

Contradictory results were reported in gastric cancer; reduction in Wnt-7a was associated with silenced CpG methylation, and Wnt-7a was shown to function to suppress epithelial–mesenchymal transition as well as tumor growth [113]. On the other hand, Wang and colleagues showed that miR-127 targeted Wnt-7a mRNA, and miR-127 was downregulated in gastric cancer [114]. These results mean that Wnt-7a could be either a tumor suppressor or promoter in gastric cancer.

In brief, the current results basically support the idea of Wnt-7a as a tumor suppressor in CRC itself; the same trends were observed in non-small cell lung cancer, renal clear cell carcinoma, and a sub-population of breast cancer. On the other hand, the trend is unclear in gastric cancer at the current moment.

### 2.6. Wnt-11

Human Wnt-11 cDNA was first cloned in 1998 [115], and another group reported it with potential corrections on three amino acid residues [116]. In normal tissues, Wnt-11 was reported to promote proliferation and migration of small intestinal epithelial cells [117] and was later shown to work synergistically with Wnt-5a in post-mesoderm anterior–posterior axis elongation in mammalian development [118].

It was just a decade ago that the first study suggested the possible involvement of Wnt-11 in CRC [119]. This is the first finding suggesting the involvement of Wnt-11 in CRC progression. In the study, the authors demonstrated a significant increase in Wnt-11 mRNA expression in primary tumor tissues, in addition to CRC cell lines, although the molecular mechanism was not clear at this point. Several years later, a more mechanistic study was published. A potential role of Wnt-11 as a tumor promoter was further reinforced by a very recent study [120]. These are cell line-based data, but there is one study reporting the potential of Wnt-11 as a prognostic biomarker in CRC [121]. In addition, elevated Wnt-11 level by a disulfide isomerase family protein, AGR2 [122]. Most recently, a study analyzing clinical data as well as immunohistochemical analyses showed that Wnt-11 was associated with poor prognosis of liver metastasis in CRC and pancreatic ductal adenocarcinoma [123]. On top of that, a Wnt-11 knockdown experiment demonstrated that Wnt-11 is responsible for immunosuppressive activity through the reduction in CD8+ cytotoxic T-cells, resulting in resistance to anti-PD-1 therapy. The receptor for Wnt-11 was not investigated in this study. However, this study also showed that calmodulin kinase II inhibition reverted the efficacy of anti-PD-1 in a mouse model of liver metastasis. The immunosuppressive activity of Wnt-11 was mediated by calmodulin kinase II phosphorylation.

Still, limited information is available, and further investigation of Wnt-11 in CRC would be beneficial to advance the field. In other solid tumors, Wnt-11 was first reported to be involved in breast, prostate, and CRC cell line migration [124]. Overexpression of Wnt-11 was shown to exert tumor-promoting activities in cervical [125], pancreatic [126], melanoma [127], and breast cancer [108,128] invasion.

Although some studies reported the anti-tumor effect of Wnt-11 in hepatocellular carcinoma [129], Wnt-11 appears to be a pro-tumorigenic factor across different solid tumors, including CRC overall.

## 3. Wnt-5a Receptors (ROR1/2) in Wnt/PCP Pathway

Both ROR1 and ROR2 are known single-transmembrane receptors that were originally discovered in 1992 [130]. In 2008, Kipps and colleagues discovered ROR1 as a highly expressed leukemia-associated surface antigen, and more importantly, they demonstrated that Wnt-5a could bind ROR1 [131]. Soon, the Nusse lab demonstrated that the tyrosine kinase activity of ROR2 is necessary to mediate Wnt-5a signaling [132].

The initial report demonstrated that ROR1 expression is a predictive biomarker for a shorter overall survival of CRC patients [133]. Therefore, Wnt-5a to ROR1 signaling can possibly explain one non-canonical Wnt signaling pathway involved in CRC progression. Although a few studies have focused on long non-coding RNA ROR1-AS1 and ROR1 regulation, three papers were retracted. Thus, I am not sure if ROR1-AS1 is at least directly involved in Wnt-5a/PCP signaling. At least the Wnt-5a-ROR1 axis appears to be one pathway being recognized in leukemia chemotaxis [134,135] and clinical response [136]. As represented by [137], the Phase I clinical trial of crimtuzumab (ROR1-blocking antibody) was carried out to investigate the clinical safety of the ROR1 signal inhibitor in chronic lymphocytic leukemia. Furthermore, a pre-clinical mouse xenograft model combining patient-derived breast tumors demonstrated the efficacy of crimtuzumab as a potential combination therapy drug with paclitaxel in breast cancer [138]. There are only seven clinical trials investigating crimtuzumab so far, and there is no crimtuzumab trial in CRC. Two out of seven studied solid tumors were looked into in this review (both were in combination with microtubule-targeting drugs); the combination with docetaxel (NCT05156905) was terminated, but the other trial combined with paclitaxel (NCT02776917) was completed [139]. In the paper, zilovertamab instead of crimtuzumab appears, but both are the same (ROR1 antibody). As this phase Ib study showed that patients were well-tolerated, phase II trials with a larger cohort may be expected. However, the study design in CRC would be different from breast or prostate cancers, as neither paclitaxel nor docetaxel is a part of standard regimens in CRC management. I described the possible reason why these drugs may not work well in CRC in a recent review [140].

The role of the Wnt-5a-ROR1 axis in CRC may still be debatable because there are only a few studies reporting the involvement of the Wnt-5a/ROR1 axis in CRC [133,141]. In addition, I spent enough space in the previous Section 2. There are significantly higher numbers of studies suggesting the tumor-suppressive effect of Wnt-5a in CRC (for example, [65,81,82,83,84,85]). Overall, the role of the Wnt5a-ROR axis in CRC is not well-defined at the current moment.

ROR2 was initially reported to inhibit canonical Wnt/β-catenin signaling [132,142,143]; thus, the Wnt-5a/ROR2 signal may function as a tumor-suppressive signal. Supporting these studies, epigenetic downregulation of ROR2 expression was indeed reported in both CRC cell lines and primary tumor samples [144,145]. However, other studies reported the correlation between high ROR2 expression levels and poor prognosis in CRC [146]. In cellular-level and in vivo studies on renal carcinoma [147] and melanoma [148], RNAi of ROR2 was shown to suppress tumor growth.

Thus, further studies will be necessary to achieve a better understanding of the Wnt-5a/ROR2 axis in CRC. The tumor-suppressive effects of Wnt-5a might still be mediated by the Wnt-5a/ROR2 pathway, yet there is little concrete evidence to support the molecular mechanisms. Although RNAi at the cellular level should be fine, knockout mice of ROR1 [149] or ROR2 [150] are dead after birth due to respiratory dysfunction or are neonatally lethal, respectively. Therefore, the use of conditional knockout systems would be essential. I will discuss these spatio-temporal knockout issues at the end of this section and in Section 4.

When we look at the receptors, induction of ROR1 expression appears to be more frequently reported than ROR2 in a variety of cancers [151]. Most recently, the hypoxia regulation of ROR1 and ROR2 expression was reported by Robles-Flores’ group [152]. In this study, the authors combined CRISPR-Cas9-based knockout of ROR1 or ROR2. The study is very notable because it showed the different proximal promoter region structures of ROR1 and ROR2 (up to 2kbp from the transcription initiation site). ROR2 has five, but ROR1 has only one hypoxia-responsive element. Indeed, ROR2 expression was induced but not ROR1 upon hypoxia. ROR2 KO was slightly more effective at halting CRC cell sheet migration than ROR1 KO. Strikingly, the ROR2 KO xenograft showed no detectable liver metastasis [152].

Another layer of complexity is that ROR1-ROR2 could form hetero-oligomers, making the clarification of ROR signaling more challenging. For example, one of the first studies in leukemia indicated the induction of chemotaxis and cell proliferation through the heterooligomerization of ROR1 and ROR2 upon Wnt-5a treatment [134]. It is not clear if the significant level of ROR1-ROR2 heterooligomerization occurs in CRC.

In summary, there are a few important questions to be clarified in CRC in regard to ROR receptors: (i) which is the major receptor for non-canonical Wnt in CRC—ROR1 or ROR2; (ii) tumor-stage specific expression of ROR1 or ROR2 in CRC; and (iii) structural and biochemical analyses of ROR1 and ROR2.

At the end, I would like to point out that Wnt-7a and Wnt-11 have not been well-characterized as ligands for ROR1/ROR2 receptors, compared to Wnt-5a; although Wnt-11/ROR2 signaling was at least acknowledged in cancer (breast cancer) in a paper cited in Section 2.5 [128]. Very recently, an endothelial-specific conditional knockout mouse study indicated that Wnt-7a was necessary for angiogenesis through ROR2 receptor [153]. A dominant-negative ROR2 receptor study in zebrafish showed decreased gastrulation convergence and extension movement of developing tissue cells involved Wnt-11 signaling [154]. Wnt-7a and Wnt-11 might mainly rely on Wnt/Ca^2+^ pathway.

## 4. Conclusions

Unlike canonical Wnt signaling, which stabilizes β-catenin, understanding the precise role of non-canonical Wnt signaling in tumorigenesis is more complicated. Here, I tried focusing on CRC that does not carry APC or β-catenin mutations (Figure 1); that is, because those patient populations are likely to develop CRC via a relatively uncommon mechanism. As the gatekeeper, APC tumor suppressor is not broken; there must be other signatures that may not show up well in the cBioportal data. I think it might be reasonable to take a look into non-canonical Wnt signaling, because the past two decades of research, including clinical trials, have dramatically improved the clinical outcomes of CRC, yet relatively younger populations and a small fraction of patients have not benefited from the progress. Some of those may fit in the population without the canonical Wnt/β-catenin pathway alteration. Nevertheless, it is clear to me that the non-canonical Wnt pathway in CRC has many obstacles in answering questions.

### 4.1. Challenge #1: Controversial Results Associated with Non-Canonical Wnts

More or less, we can see the opposite results in the effect of non-canonical Wnt (Wnt-5a, -7a, and -11 in this review) in both CRC and other solid tumors. As discussed in [17], these controversies may be the products of the multiple layers of signaling mechanisms, including the crosstalk with the canonical Wnt pathway. In Wnt-5a particularly, a sharp contrast and controversy are seen between CRC and other solid tumors (Section 2.1, Section 2.2, Section 2.3 and Section 2.4). It is likely that there are some tumor type-specific contexts. Thus, clearer and more comprehensive catalogs of non-canonical Wnt ligands as well as receptors in CRC would be essential to solve issues step-by-step.

I also want to point out one potential issue associated with any studies using CRC cell lines; as pointed out in Section 2.4, pretty much all CRC cell lines carry some mutations in APC. Therefore, any studies employing cell lines carrying truncated APC may make the data interpretation potentially more difficult due to accumulation of β-catenin. The choice of CRC cell lines needs to be carefully done. In this regard, the Robles-Flores lab utilized a great system (112CoN for non-malignant and RKO for normal canonical Wnt signaling; [152]).

### 4.2. Challenge #2: Receptor Variations

In addition to its potential interactions with canonical Wnt receptors, non-canonical Wnt signaling pathways also have a variety of receptors [14]. Although Ryk (receptor related to tyrosine kinase) has also been known as another single-transmembrane receptor kinase in the non-canonical Wnt pathway, there are much fewer studies linking Wnt-5a and Ryk compared to ROR1/ROR2. Ryk was originally discovered back in the early 1990s [155,156], followed by a study suggesting its involvement in the transforming ability of fibroblasts and tumorigenicity [157]. Other studies reported Ryk as an axon guidance signaling receptor [158], and also showed to play an important role in proliferation and reactive oxygen species suppression by Wnt-5a [159]. It was a little over 10 years before research clearly pointed out Ryk as a critical signaling receptor for Wnt-5a in glioma [160]. Around the same time, Ryk knockdown was shown to resensitize melanoma to B-Raf inhibitor, suggesting Ryk as a potential target in clinical oncology [161]. Yet, the field seems to be premature right now.

Very interestingly, a recent study compared the structural differences between ROR2 and Frizzled (Frizzled 8) cysteine-rich domains (CRDs) using X-ray crystallography [162]. The structural comparison highlighted that the ROR2 CRD lacks a hydrophobic groove that is critical for binding of palmitoleate or cholesterol molecules to the CRD. Since non-canonical Wnt ligands still utilize Frizzled receptors, ultimately, we probably need to identify which Frizzled is predominantly utilized with ROR2 (this combination may be cancer-type-specific context). Although I do not know any perfect answers to this, Griffiths and colleagues probably showed one of the best systems to date, as their study also developed Cre recombinase-mediated conditional ROR1 and ROR2 knockout mouse embryonic fibroblasts. This system with add-back certainly allowed very precise experiments. When ROR2 deletion mutants were added back, it was clear that CRD was required to exert full capacity in Wnt-5a signaling. One interesting fact in this study was that the cytoplasmic portion of ROR2 was almost not required to restore the full signaling activity. Now, we may need to understand the significance of the cytoplasmic domains and residues (potential phosphorylation sites) of ROR2 in the non-canonical Wnt pathway.

There was another study before the above study that compared CRDs between ROR and Ryk [163]. In this study, based on X-ray crystallography, the group compared CRDs as well as the Wnt-inhibitory factor domain in *Drosophila* orthologs of ROR and Ryk. No lipid-binding motif was found in this study as well.

### 4.3. Challenge #3: Technical Difficulties in Conducting Biochemical Assays with Wnt Ligands

Non-canonical Wnt ligands, Wnt-5a, Wnt-7a, and Wnt-11, are approximately 38, 35.5, and 39.2 kDa, respectively. All molecular weights are similar and small, and recombinant proteins have been produced using *Escherichia coli*. To date, no structural information of Wnt-11 has been deposited in the Protein Data Bank. Wnt-5a’s affinity (Kd values) to a variety of Frizzles was very recently reported [164], although there are no known studies that performed biochemical measuring of the binding affinity using purifed proteins. The study utilized a system called NanoBiT/BRET, essentially the combination of luciferase and energy transfer between NanoLuc luciferase and a fluorescent protein. Thus, it could evaluate Wnt and receptor interactions [164].

Because of post-translational modifications of Wnt-5a, Wnt-7a, and Wnt-11 through carbohydrates and lipids, we probably require at least an insect cell system (such as *Drosophila* S2 cells) to produce the complete forms of those Wnt molecules. Still, strictly speaking, in the study cited in Section 4.2 [163], it was shown that *Drosophila* Wnt5 could bind to both ROR and Ryk without an acyl chain. I am not sure if this is a *Drosophila*-specific phenomenon. Further studies in mammalian systems would be desired. The current idea is that a few of the Frizzled receptors may function as a co-receptor with ROR2, as clearly demonstrated by using, i.e., blocking Frizzled2 with a monovalent antibody-binding fragment (Fab) could almost completely inactivate the Wnt-5a-induced signaling [162]. It should be noted that the dimerization of Frizzled could induce signaling, and thus the authors developed Fab [162]. The study was very carefully conducted.

### 4.4. Challenge #4: Cancer-Associated Fibroblasts (CAFs)

The review article by Plikus and colleagues provided a great overview of fibroblasts in healthy and diseased tissues, as well as a few organ-specific fibroblasts [165]. It is very interesting to know that a small subset of fibroblasts (collagen triple helix repeat-containing 1 (CTHRC1) gene-positive) was the major source of Wnt-5a, thereby promoting epithelial–mesenchymal transition in CRC [166]. Prior research showed that CTHRC1 antibody might be a useful biomarker in CRC [167], and, indeed, CTHRC1 was shown to promote CRC proliferation and invasion via Wnt/PCP signaling, one of the non-canonical Wnt pathways [168]. Thus, it is likely that CTHR1 is either directly or indirectly involved in CRC progression.

**Figure 3:**
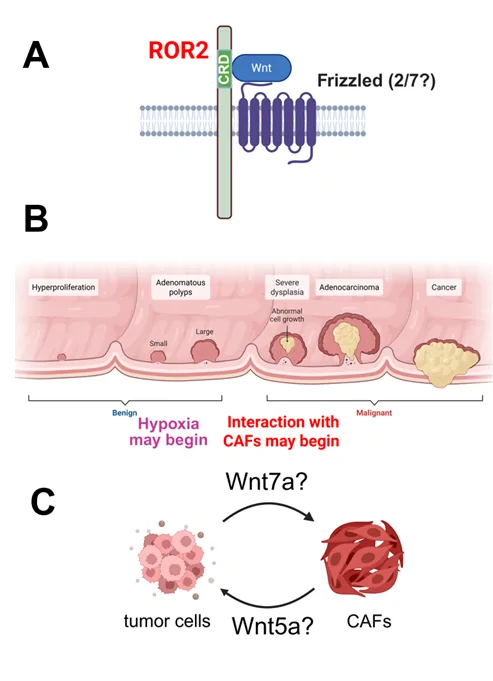
The schematic illustrations based on some of the very notable recent studies (created in BioRender. Kita, K. (2026), https://BioRender.com/kvrta2f). (**A**) The relationship between ROR2 and Frizzled. Although the ROR2 CRD domain does not have a lipid-binding groove; Frizzled’s high-affinity CRD may assist ROR2 in effectively binding Wnt-5a [162]. (**B**) A simple summary of CRC progression and possible beginning of hypoxia and interaction with CAFs. (**C**) Possible paracrine mechanisms between CRC cells and CAFs. Hypoxia-induced CAF activation and Wnt-5a production were very recently demonstrated (see Section 4 [86]).

Although this is the case in breast cancer, one study reported CAF activation by tumor cell-derived Wnt-7 [169]. This study was conducted using an in vivo model, and aggressive forms of tumors activated CAFs more in a TGF-β-dependent manner. As introduced in Section 4.3, Wnt-7a helped ovarian cancer progression [107,109]. It is possible that a paracrine activation or suppression between tumor cells and CAFs mediated by a few non-canonical Wnt ligands (Figure 3B).

### 4.5. Challenge #5: Cancer-Stage-Dependent Change in the Role of Non-Canonical Wnt Signaling

This idea is coined by a very recent study reporting the induction of ROR1 and ROR2 by hypoxia in CRC cells [152]. Hypoxia in CRC during tumor progression would trigger multiple layers of events that are associated with tumor heterogeneity [170]. When the tumor grows, the availability of oxygen inside the tumor tissues decreases, and subsequent events will occur. Most recently, the Kikuchi lab reported that hypoxia-induced activation of CAFs caused Wnt-5a secretion in CRC [86]. This is a very timely study in the field, and the authors developed a conditional Wnt-5a knockout mouse system, then used it to induce CRC by the azoxymethane/dextran sodium sulfate treatment [86]. In this way, the mouse system probably mimics the condition in human CRC development. More importantly, Wnt-5a was induced in this system, and the Cancer Gene Atlas data analysis demonstrated that Wnt-5a is the primary non-canonical Wnt induced in tumors. Further experiments in this study pointed out that Wnt-5a induced in this experimental CRC system was CAF-origin. The study also utilized single-cell RNA-seq to address tumor heterogeneity [86]. Overall, this study may begin unveiling the tense controversies about the role of Wnt-5a in CRC.

Increasing numbers of studies have shown that Wnt-5a is more likely to be upregulated in a variety of solid tumors but CRC-based on more papers reporting the lower expression of Wnt-5a or the promoter methylation in CRC [65,81,82,83,84,85], decreased expression of Wnt-5a may be one of the factors that play a role in the progression of CRC that does not harbor APC or β-catenin mutations.

As I have pointed out, the presence of the two isoforms of Wnt-5a [90] might hold the key to understanding the controversial effects of Wnt-5a in CRC, as well as in other cancers. At least in CRC, one study demonstrated possibly distinct roles of the two isoforms [92]. The other critical areas would be (i) the signaling pathway mediating the tumor-suppressive effect of Wnt-5a (what is the main receptor?) and (ii) the role of Wnt-5a on CAF. As briefly discussed in Section 2.5—the role of Wnt-5a receptors (ROR1/2) in the Wnt/PCP pathway—we definitely do not have enough information about the receptors for Wnt-5a, specifically in CRC, although the Wnt-5a/ROR2 pathway might explain the role of Wnt-5a either as a tumor suppressor or promoter. Regarding Wnt-5a and CAF activation, so far, only one study [70] has clearly pointed out CRC progression by Wnt-5a. Therefore, it is too early to draw conclusions CAF as the source of Wnt-5a in CRC yet. Still, the structural study using ROR1/ROR2 conditional knockout fibroblasts [162] and recent hypoxia-related studies [86,152] have clearly begun making breakthroughs to understand the role of the non-canonical Wnt/PCP pathway in CRC. Figure 3 summarizes a possible mechanism based on these as well as other studies cited in Section 4.4.

Bueno and colleagues discussed the role of Wnt-5a in the tumor microenvironment [171], and it is an excellent read. In brief, it is clear to me that the role of non-canonical Wnt in CRC will require more attention to elucidate the controversial results reported in earlier days. Future studies will be built around conditional knockout mouse model systems for non-canonical Wnt molecules and receptors, as discussed in the challenges #3~5 in Section 4.

## Data Availability

All data are included in the manuscript.
